# Newborn metabolomic perturbations associated with prenatal tobacco smoke exposure and early birth

**DOI:** 10.1038/s43856-026-01534-5

**Published:** 2026-04-01

**Authors:** Paula-Dene C. Nesbeth, Xiajie Lyu, Anne L. Dunlop, Youran Tan, Dana Boyd Barr, Volha Yakimavets, Parinya Panuwet, Mengyuan Ren, Stephanie M. Eick, Blake R. Rushing, Susan L. McRitchie, Susan Sumner, P. Barry Ryan, Elizabeth J. Corwin, Dean P. Jones, Donghai Liang

**Affiliations:** 1 Gangarosa Department of Environmental Health, Rollins School of Public Health, Emory University, Atlanta, GA USA; 2 Department of Gynecology and Obstetrics, School of Medicine, Emory University, Atlanta, GA USA; 3 Department of Nutrition, University of North Carolina at Chapel Hill, Chapel Hill, NC USA; 4 Nutrition Research Institute, University of North Carolina at Chapel Hill, Kannapolis, NC USA; 5 School of Nursing, Columbia University, New York, NY USA; 6 School of Medicine, Emory University, Atlanta, GA USA

**Keywords:** Biomarkers, Metabolomics, Epidemiology, Metabolomics

## Abstract

**Background:**

Exposure to tobacco smoke during pregnancy is an established risk factor for early birth including early term birth (ETB) and preterm birth (PTB). However, the underlying molecular mechanisms are minimally understood. In this study, we aimed to characterize the newborn metabolomic associations with early pregnancy maternal tobacco exposure biomarkers and early birth in 269 mother-child pairs in the Atlanta African American Maternal-Child Cohort (2016–2020).

**Methods:**

Established tobacco exposure biomarkers, cotinine and trans-3′-hydroxycotinine (3HC), were measured in maternal urine samples collected between 8–14 weeks of gestation. Newborn dried blood spots were collected for high-resolution metabolomics profiling. Metabolome-wide association studies and pathway enrichment analyses were conducted to determine metabolomic signals and pathways associated with tobacco exposure biomarkers, ETB, and PTB.

**Results:**

We show that biopterin metabolism is a significantly enriched pathway for all exposures and outcomes. Both tobacco exposure biomarkers are associated with riboflavin metabolism. The metabolites riboflavin and 5-hydroxytryptophan are associated with all exposures and outcomes.

**Conclusions:**

Taken together, these findings demonstrate that the newborn metabolome is altered by prenatal tobacco exposure and that these alterations are associated with elevated risks of early birth. Furthermore, perturbation in biopterin metabolism is a potential mechanism linking maternal tobacco exposure to early birth.

## Plain language summary

Maternal tobacco exposure during pregnancy increases the risk of babies being born too early, but the biological reasons for this are not well understood. In this study, we analyzed tiny chemical signals called metabolites in newborn blood samples and compared them with measures of tobacco exposure during early pregnancy and birth timing. We found that tobacco exposure is linked to specific changes in the newborn’s chemistry that are also associated with early birth. These changes point to a biological pathway that may help explain how tobacco affects pregnancy. Our findings improve understanding of how tobacco exposure influences early delivery and may help guide future prevention efforts for pregnant women with high exposure.

## Abstract (web summary)

Nesbeth et al. use metabolome-wide association studies to identify newborn metabolic profiles associated with prenatal tobacco exposure and early birth. Results reveal changes in biopterin metabolism as a potential link between prenatal tobacco exposure and early birth.

## Introduction

Smoking during pregnancy is one of the most easily preventable risk factors for adverse birth outcomes, including early term birth (ETB; 37–38 completed weeks of gestation) and preterm birth (PTB; <37 weeks of gestation)^1–3^. These adverse health outcomes have major health implications for offspring during their life course, including increased risks of adverse neurodevelopmental outcomes, cardiovascular disease, and cancer^4–6^. African American women are disproportionately affected by PTB, impacting approximately 14.4% of births in this group compared to 9.1% for White women in the United States (US)^7^. Although reports show that the prevalence of smoking during pregnancy is lower among Black women compared to White women, Black Americans have higher second-hand tobacco smoke exposure^8,9^. Non-smoking African American women exposed to tobacco smoke are therefore exposed to tobacco-derived toxicants like smokers. Few studies have evaluated tobacco smoke exposure and its relationship with early births, specifically in this population^10^.

Tobacco exposure can be estimated by measurement of specific nicotine-derived metabolites in biospecimens. Cotinine and trans-3′-hydroxycotinine (3HC) are major nicotine metabolites and established biomarkers of tobacco exposure in human biological samples^11,12^. While the epidemiological link between tobacco exposure and higher risk of early birth outcomes is well established, the exact biological mechanisms connecting tobacco exposure to early birth outcomes are less clear^13^. Postulated mechanisms include elevated inflammation and oxidative stress that lead to abnormal placental development and restricted fetal growth^14,15^. Metabolites with antioxidant properties, for example, may be perturbed due to tobacco exposure, and these perturbations may be contributors to early birth.

Our research group has leveraged high-resolution metabolomics (HRM) to identify metabolic perturbations and potential biomarkers associated with other environmental exposures and adverse birth outcomes in a well-characterized population of African American women and their children. HRM is a combined analytical and computational approach to detect and characterize thousands of small molecules and their associations with an exposure and/or health outcome, providing a comprehensive view of biological responses^16–23^. To our knowledge, only a small number of studies have examined whether maternal tobacco exposure during pregnancy alters the fetal-placental unit or infant circulating metabolites^24–28^. Whether these metabolic changes in offspring are also linked to early birth has not been elucidated. Compounding this knowledge gap is a paucity of research studies generally focused on pregnant African American women and their newborns, despite the observed disparities in adverse birth outcomes^20^.

In this study, we use a well-established HRM approach to analyze untargeted metabolites in newborn dried blood spot (DBS) samples and conduct metabolome-wide association studies (MWAS) to identify metabolites altered in newborns that are associated with maternal urinary cotinine and 3HC concentrations (exposure) and early births (outcomes) in an established cohort of African American mother-child pairs^16,23,29,30^. Our MWAS results reveal that riboflavin and 5-hydroxytryptophan are associated with both prenatal tobacco exposure and early birth outcomes. Pathway enrichment analyses show perturbation in biopterin metabolism as a potential intermediate pathway linking prenatal tobacco exposure to ETB and PTB in this population.

## Methods

### Study population

The participants in this study are from the larger Atlanta African American Maternal-Child Cohort. This is a prospective cohort study of US-born African American pregnant women and their children residing in Atlanta, GA, aimed at identifying the distinct attributes driving adverse maternal and child health disparities for African Americans. Protocols for the cohort have been previously published^31,32^. Briefly, pregnant women were recruited from Emory Midtown Hospital and Grady Memorial Hospital in Atlanta, GA, USA. Inclusion criteria consisted of self-identification as African American or Black, born in the US, between 18 and 40 years old, with a singleton pregnancy between 8 and 14 weeks of gestation, and no chronic medical conditions. Participants in this analysis had delivery due dates between 2016 and 2020. Pregnant women provided written informed consent for their participation in the study through the delivery admission and for the Georgia Department of Public Health Laboratory to provide us with the filter paper left over from their newborn’s blood spot collection for newborn metabolic screening. This study was approved by the Emory University Institutional Review Board (approval reference number 68441) and conducted in accordance with the Declaration of Helsinki.

Maternal data was collected through a combination of methods during the early pregnancy clinic visit (8–14 weeks of gestation). Demographic characteristics were self-reported and captured by a questionnaire. Clinical conditions (e.g., parity and prenatal body mass index (BMI)) were abstracted from medical records. Tobacco, marijuana, and alcohol use in the month prior to the study visit or anytime in pregnancy were abstracted from medical records and by self-report, respectively. Our analytic sample consisted of 269 mother-child pairs who had early pregnancy maternal urine samples available to determine cotinine and 3HC concentrations and had newborn metabolomics profiling conducted using the DBS samples.

### Maternal tobacco exposure biomarkers

Spot urine samples were collected during early pregnancy and were analyzed using a previously published method^33^. Briefly, 200 µL urine was spiked with isotopically labeled internal standards and enzymatically hydrolyzed to liberate the conjugated analytes before extraction using solid-supported liquid-liquid extraction with ethyl acetate elution. After concentration of the extract, the target analytes were analyzed using liquid chromatography-positive electrospray ionization-tandem mass spectrometry (LC-MS/MS) with multiple reaction monitoring of target ion transitions. Isotope dilution calibration was used to quantify the analytes. The limit of detection (LOD) for cotinine was 1.25 ng/mL. The LOD for 3HC was 1.25 ng/mL. Accuracy was determined by the National Institute of Standards and Technology (NIST) reference samples, which showed results between 86.8% and 95.3%. The method was validated by participating in the German External Quality Assessment Scheme proficiency testing scheme (http://gequas.de). If concentrations of the tobacco exposure biomarkers were below the LOD, they were imputed by dividing the LOD by √2^34^. To account for the variable urine dilution, creatinine concentrations in the urine samples were also measured. The urinary cotinine and 3HC concentrations were adjusted using creatinine concentrations for each sample (biomarker concentration/creatinine concentration × 100) prior to statistical analyses.

### Birth outcomes

Gestational age at birth was abstracted from medical records and determined from the estimated date of conception to the date of delivery. The estimated date of conception was established at the prenatal clinic visit by self-reported last menstrual period and/or ultrasound^35^. Gestational age at birth was defined in alignment with American College of Obstetricians and Gynecologists guidelines: PTB (22 to <37 weeks), ETB (37–38 weeks), and full-term (≥39 weeks)^3^.

### Untargeted high-resolution metabolomics

The collection of newborn DBS is considered routine for medical screening and public health surveillance^36,37^. Newborn DBS samples were collected using a standardized protocol by trained hospital nursery personnel from a heel stick within 24–48 h of birth for metabolomic profiling. The standardized protocol involved cleaning the heel with 75% isopropanol, using a sterile 2.5 mm lancet to obtain the blood sample, and saturating each circle on a standard Guthrie card^36^. The card specimens were transported to the Georgia Department of Public Health Laboratory on the same collection day and kept in a walk-in refrigerator (2–8 °C) without a desiccant for up to 3 months. The DBS samples were then moved to our laboratory for storage at −80 °C using gas-impermeable bags with a desiccant until the assay^36^. The study obtained single 15-mm punches for analysis, and an extra set of 15-mm punches was collected from adjacent filter paper portions with no blood from the same Guthrie cards to use as blanks.

Untargeted HRM profiling was conducted using established methods by the North Carolina HHEAR Hub in the University of North Carolina Nutrition Research Institute, North Carolina Research Campus (Kannapolis, NC, USA)^16,29,30^. Briefly, DBS samples were extracted by sonication in 1 mL of ice-cold methanol containing 500 ng/mL L-tryptophan-d5, and the supernatant was transferred to low-bind microfuge tubes. All supernatant aliquots were pooled to make a quality control study pool. Study samples and study pool aliquots were dried by a vacuum concentrator and reconstituted with 95:5 water:methanol (v/v) prior to analysis by ultra-high-performance liquid chromatography coupled with high-resolution mass spectrometry. Method blanks were created by extracting all matched blank cards using the same extraction procedure and pooling the extracts together. NIST reference plasma samples were also included as external quality control samples. Detailed methods are described in our previous publications^16,23^.

Untargeted metabolomics data were acquired using a Q Exactive™ HF-X Hybrid Quadrupole-Orbitrap Mass Spectrometer (Thermo Fisher Scientific, San Jose, CA). Separation of metabolites was carried out using a HSS T3 C18 column (2.1 × 100 mm, 1.7 μm) (Waters Corporation, Milford, MA) after a 5 µL injection of the sample. Progenesis QI (version 2.1, Waters Corporation) was used for data preprocessing, which included peak picking, alignment, and normalization^38^. To minimize batch effects, signals that significantly differed by ANOVA with a false discovery rate (FDR) value of *q* < 0.05 amongst the three running batches were excluded from further analysis. The metabolomics data were filtered to include only signals identified in at least 20% of all participant samples and log_2_-transformed prior to statistical analysis to normalize positive skewness.

### Statistical analysis

Descriptive statistics were calculated to assess distributions of demographics in our analytic sample. For continuous variables, this included mean ± standard deviation (SD), and frequency (*n*) and percentage (%) for categorical variables. Four MWAS were conducted to identify metabolomic signals associated with creatinine-adjusted cotinine and 3HC concentrations and early birth (ETB and PTB). Covariates included in analysis were identified by a review of the literature and a directed acyclic graph (Supplementary Fig. 1). For tobacco exposure MWAS, multivariable linear models were used to evaluate the associations between maternal urinary cotinine (Model 1) and 3HC (Model 2) concentrations and intensity of each newborn metabolomic signal.

1 $${\log }_{2}({\mathrm{Intensity}}) = 	 \, {\upbeta }_{0}+{\upbeta }_{1}{\mathrm{Cotinine}}+{\upbeta }_{2}{\mathrm{Neonatalsex}} \\ 	 + {\upbeta }_{3}{\mathrm{Maternalage}}+{\upbeta }_{4}{\mathrm{BMI}}+{\upbeta }_{5}{\mathrm{Education}} \\ 	 + {\upbeta }_{6}{\mathrm{Parity}}+{\upbeta }_{7}{\mathrm{Alcohol}}+\upepsilon$$

2 $${\log }_{2}\left({\mathrm{Intensity}}\right) = 	 \, {\upbeta }_{0}+{\upbeta }_{1}3{\mathrm{HC}}+{\upbeta }_{2}{\mathrm{Neonatalsex}} \\ 	 + {\upbeta }_{3}{\mathrm{Maternalage}}+{\upbeta }_{4}{\mathrm{BMI}} \\ 	 + {\upbeta }_{5}{\mathrm{Education}}+{\upbeta }_{6}{\mathrm{Parity}}+{\upbeta }_{7}{\mathrm{Alcohol}}+\upepsilon$$

For each tobacco biomarker model, intensity represents each metabolomic signal, while *β*_0_ is the intercept. The regression estimates for creatinine-adjusted cotinine (in Model 1) and creatinine-adjusted 3HC (in Model 2) are denoted as *β*_1_. The covariates included for both tobacco exposure biomarkers MWAS were neonatal sex (categorical), maternal age (continuous), BMI (continuous), education (categorical), parity (categorical: no prior birth [nulliparous] or ≥1 prior births [parous]), and alcohol use (categorical) and their regressions estimate coefficients are represented by *β*_2–7_. *ϵ* denotes random error.

To identify metabolomic signals that may be linked to adverse birth outcomes, logistic regression models were used for each early birth outcome with full term birth as the reference group (Models 3 and 4). FDR correction for multiple testing was performed using the Benjamini-Hochberg method^39^. Statistical analyses were conducted in R (version 4.4.2, https://www.R-project.org).

3 $${{\mathrm{ln}}}\left(\frac{P({{\mathrm{ETB}}})}{1-P({\mathrm{ETB}})}\right) = 	 \, {\upbeta }_{0}+{\upbeta }_{1}{\log }_{2}({\mathrm{Intensity}}) \\ 	 + {\upbeta }_{2}{\mathrm{Neonatalsex}}+{\upbeta }_{3}{\mathrm{Age}}+{\upbeta }_{4}{\mathrm{BMI}} \\ 	 + {\upbeta }_{5}{\mathrm{Education}}+{\upbeta }_{6}{\mathrm{Parity}}+{\upbeta }_{7}{\mathrm{Alcohol}}$$

4 $${{\mathrm{ln}}}\left(\frac{P\left({{\mathrm{PTB}}}\right)}{1-P\left({{\mathrm{PTB}}}\right)}\right) = 	 \, {\upbeta }_{0}+{\upbeta }_{1}{\log }_{2}({\mathrm{Intensity}})+{\upbeta }_{2}{\mathrm{Neonatalsex}} \\ 	 + {\upbeta }_{3}{\mathrm{Age}}+{{{\rm{\beta }}}}_{4}{\mathrm{BMI}}+{\upbeta }_{5}{\mathrm{Education}} \\ 	 + {\upbeta }_{6}{\mathrm{Parity}}+{\upbeta }_{7}{\mathrm{Alcohol}}$$

### Chemical annotation

To confirm the chemical identity of the significant metabolomic signals, we used an in-house experimental standards library (IESL) including endogenous and exogenous metabolites^16,23^. Confidence in metabolite annotations of metabolomic signals was determined by ontology level (OL), which incorporates chemical characteristics for each metabolite, including retention time (RT), exact mass (MS), MS/MS fragmentation pattern, and isotopic ion pattern. OL1 metabolites were matched to the IESL by RT (±0.5 min), MS (< 5 ppm), and MS/MS (similarity score >30). OL2a metabolites matched by RT and MS. Metabolites matched to metabolomic signals by MS and MS/MS to the IESL but outside the RT window were labeled as OL2b. OL1 and OL2a metabolites were considered as high confidence matches^16,23^. These OLs correspond to level 1 matches under the definitions of the Metabolomics Standards Initiative^40^. In cases of multiple matches for the same metabolite across OL categories, only the OL1 annotation was reported.

### Pathway enrichment analysis

Pathway enrichment analyses were performed using *mummichog* (version 2.7), which is a computational algorithm for predicting metabolites and their biological pathways from HRM data^41^. For each MWAS, metabolomic signals with raw *p* values < 0.05 were selected for pathway enrichment analysis. Metabolic pathways identified by *mummichog* with *p* value < 0.05 and ≥10% of metabolites in the pathway were considered significant.

### Meet-in-the-middle analysis

We conducted multiple *meet-in-the-middle* analyses to investigate whether the changes in the newborn metabolome mediate the link between maternal cotinine and 3HC concentrations and early birth outcomes (PTB and ETB)^16,19,20,42,43^. These analyses involved performing separate MWAS and pathway enrichment analyses for each tobacco exposure biomarker and birth outcome. Common pathways and metabolomic signals that had significant associations with both exposure and outcome were identified as potential intermediaries. We also performed a sensitivity analysis using metabolomic signals with FDR-corrected *q* values < 0.2 for metabolic pathway enrichment analysis to identify similarities in the enriched pathways associated with tobacco exposure and early birth. In this study, the *meet-in-the-middle* approach was applied as a hypothesis-generating strategy to identify shared metabolic signals and pathways associated with both prenatal tobacco exposure and early birth outcomes. Newborn DBS metabolomic profiles were not conceptualized as causal mediators acting after birth, but rather as proximal biomarkers reflecting cumulative in utero biological perturbations occurring prior to delivery^16,23^. Shared associations were interpreted as indicators of potential biological mechanisms linking prenatal tobacco exposure to early birth risk, rather than evidence of formal temporal mediation.

## Results

### Study characteristics

The characteristics of participants included in this analysis are summarized in Table 1. The mean (SD) maternal age of pregnant participants in the study was 25.6 (5.2) years, and the mean early pregnancy BMI was 29.2 (7.8) kg/m^2^. Highest education attained varied with 14.5% having less than high school education, 43.5% with a high school education, and 42% with some or more college education. Parous women comprised 58% of participants. Some participants reported tobacco (16.4%), alcohol (12.6%), and marijuana (37.2%) use in the month prior to the study visit or anytime in pregnancy. The majority of births in this analysis were full-term (56.5%), 31.2% were early term, and 12.3% were preterm. Neonatal sex was almost equal between females (51.3%) and males (48.7%). The majority of participants, including participants reporting no tobacco use, had detectable concentrations of urinary cotinine and 3HC. Geometric mean (SD) urinary creatinine-adjusted cotinine and 3HC concentrations were 15.75 (9.37) µg/g and 36.56 (9.85) µg/g, respectively, for pregnant participants with concentrations above LOD (Table 2). Descriptive statistics of cotinine and 3HC concentrations for all participants are shown in Supplementary Table 1.

**Table 1:** Characteristics of sample population from the Atlanta African American Maternal-Child Cohort, 2016-2020 (*n* = 269)

Characteristic	All participants
Age at enrollment, y	25.6 ± 5.2
Prenatal BMI, kg/m^2^	29.2 ± 7.8
Education level	
Less than high school	39 (14.5)
High school	117 (43.5)
Some college or more	113 (42.0)
Nulliparous	
Yes	113 (42.0)
No	156 (58.0)
Neonatal sex	
Female	138 (51.3)
Male	131 (48.7)
Alcohol use^a^	
Yes	34 (12.6)
No	235 (87.4)
Marijuana use^a^	
Yes	100 (37.2)
No	169 (62.8)
Tobacco use^a^	
Yes	44 (16.4)
No	225 (83.6)
Birth outcome ^b^	
Preterm	33 (12.3)
Early term	84 (31.2)
Full-term	152 (56.5)

Values are expressed as mean ± SD or *n* (%).

*Y* years, *BMI* body mass index.

^a^Use within the month prior to the study visit or anytime in pregnancy.

^b^Birth outcomes were categorized into preterm, 22 to <37 gestational weeks; early term, 37–38 gestational weeks; full-term, ≥39 gestational weeks.

**Table 2:** Maternal unadjusted and creatinine-adjusted cotinine and 3HC concentrations for participants with concentrations above LOD

	All participants (*n* = 269)	No tobacco use^a^ (*n* = 225)	Tobacco use^a^ (*n* = 44)
*Cotinine*			
Above LOD (*n*, %)	205 (76.2)	161 (71.6)	44 (100)
Unadjusted (ng/mL urine)	28.53 (8.84)	15.25 (5.94)	282.26 (7.15)
Adjusted (μg/g creatinine)	15.75 (9.37)	8.24 (6.26)	168.95 (7.22)
*3HC*			
Above LOD (*n*, %)	208 (77.3)	164 (72.9)	44 (100)
Unadjusted (ng/mL urine)	66.58 (9.47)	34.71 (6.27)	754.87 (7.21)
Adjusted (μg/g creatinine)	36.56 (9.85)	18.63 (6.42)	451.82 (7.16)

Values reported are geometric mean (geometric SD).

*3HC* trans-3′-hydroxycotinine, *LOD* limit of detection.

^a^Tobacco use in the month prior to the study visit or anytime in pregnancy.

### Newborn metabolomic signals associated with biomarkers of tobacco exposure and early birth outcomes

After data preprocessing, quality control, and data filtering, there were 6558 signals in the metabolomics dataset. After adjusting for covariates, 645 and 494 signals were significantly associated with maternal cotinine and 3HC concentrations, respectively (*p* value < 0.05). There were 671 signals associated with ETB and 1729 signals associated with PTB (*p* value < 0.05) (Supplementary Fig. 2). After FDR correction, 117 signals were linked to cotinine concentrations, while 48 signals were associated with 3HC concentrations (*q* value < 0.2). At the FDR-corrected *q* value < 0.2 threshold, we identified 52 and 1788 signals associated with ETB and PTB, respectively (Supplementary Fig. 3). The numbers of significant metabolomic signals for each combination of exposure and outcome at each significance threshold are presented in Supplementary Table 2 and Supplementary Fig. 4 (for *p* value < 0.05) and Supplementary Table 3 and Supplementary Fig. 5 (for *q* value < 0.2).

**Fig. 1:**
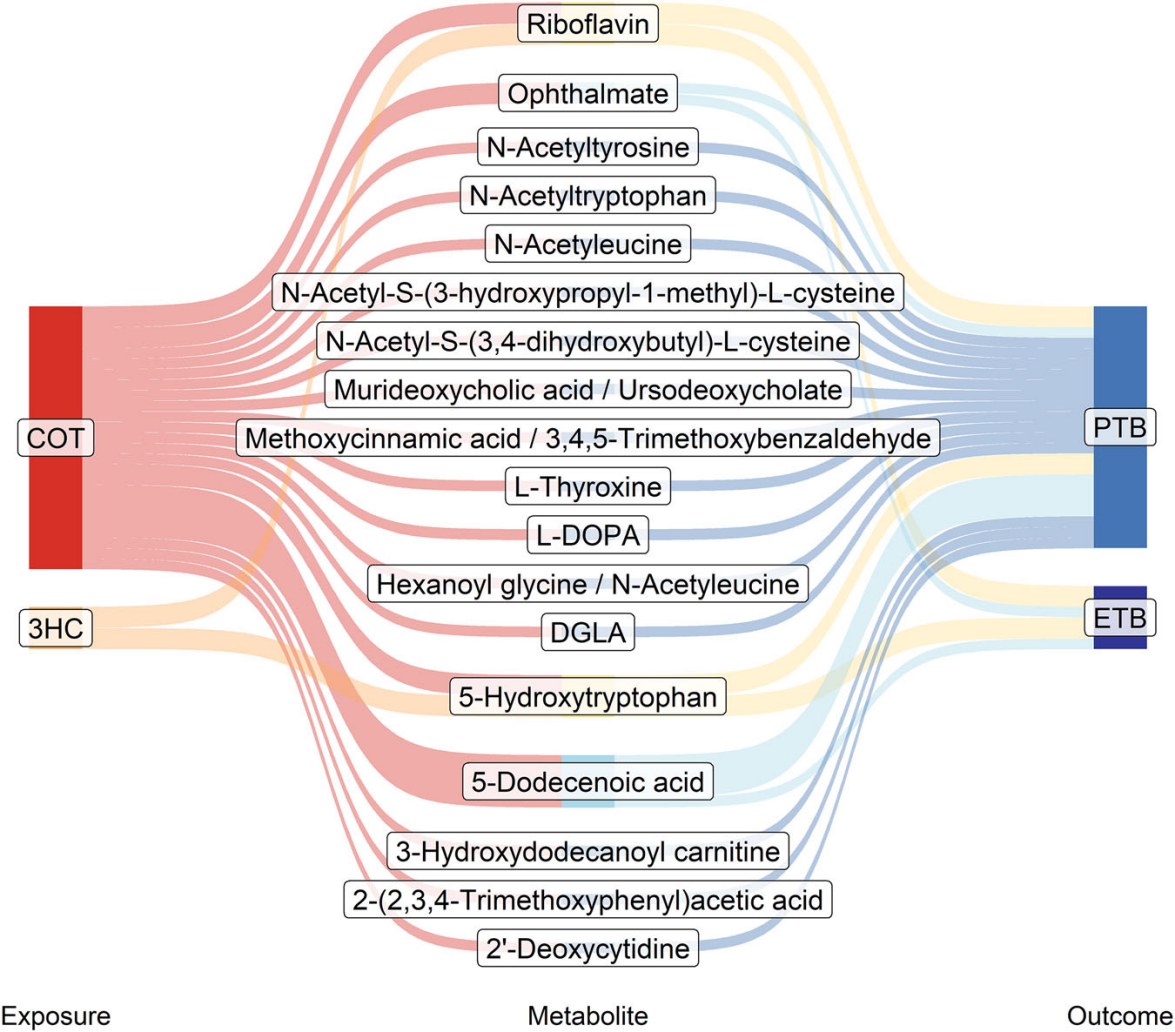
Sankey plot summarizing metabolites associated with prenatal tobacco exposure and early birth outcomes. The nodes on the left are the tobacco exposure biomarkers, cotinine and 3HC. The nodes on the right are the early birth outcomes, ETB and PTB. The flow to each metabolite represents a significant association (*p* value < 0.05) with the metabolite in the middle stage. Metabolites shown are OL1 and OL2a annotations and were significantly associated with at least one tobacco exposure biomarker and one early birth outcome. Statistical tests conducted were multivariable linear regression (for tobacco exposure biomarkers) and multivariable logistic regression (for early birth outcomes). The red and orange colors on the left-hand side represent tobacco exposure biomarkers, cotinine and 3HC, respectively. The blue and dark blue colors on the right-hand side represent PTB and ETB, respectively. Flows on the right-hand side that are colored yellow are metabolites significantly associated with all exposures and outcomes. Flows on the right-hand side that are colored light blue are associated with one tobacco exposure biomarker and both early birth outcomes. Regression coefficients and two-sided *p* values are shown in Table 3. COT cotinine, DGLA dihomo-gamma-linolenic acid, L-DOPA L-3,4-dihydroxyphenylalanine, ETB early term birth, 3HC trans-3′-hydroxycotinine, PTB preterm birth.

The annotation of the significant metabolomics signals (*p* value < 0.05) that overlapped with at least one of the tobacco exposure biomarkers and at least one early birth outcome resulted in 32 matches confirmed with high confidence: 8 as OL1, 12 as OL2a, and 12 as OL2b (Table 3, Fig. 1). Primary metabolic categories that were represented by metabolites with OL matches included amino acid and proteins, fatty acid and lipids, xenobiotics, bile acids, vitamins, and nucleosides. Two metabolites, riboflavin (vitamin B2) and 5-hydroxytryptophan, were significantly altered across all MWAS. Riboflavin was positively associated with both tobacco exposure biomarkers and early birth, while 5-hydroxytryptophan was negatively associated with the exposures and outcomes. Other metabolites of interest that were associated with at least one of the tobacco exposure biomarkers and one of the early birth outcomes were ophthalmate, 3,4-dihydroxyphenylalanine (L-DOPA), dihomo-gamma-linolenic acid (DGLA), 3-hydroxydodecanoyl carnitine, and glutamine. Lastly, thyroxine was negatively associated with cotinine and PTB. FDR-corrected *q* values for high confidence metabolite matches are presented in Supplementary Table 4. The relationships between the main metabolites significantly associated with both tobacco exposure biomarkers and both early birth outcomes (5-hydroxytryptophan and riboflavin) were further examined stratified by marijuana use (Supplementary Table 5).

**Table 3:** Chemical annotation of newborn dried blood spot metabolomic signals associated with prenatal tobacco exposure biomarkers (creatinine adjusted) and early birth outcomes

Metabolite^a^	*m*/*z*	rt (min)	Category	Cotinine^b^		3HC^b^		ETB^c^		PTB^c^	
				*β*	*p*	*β*	*p*	*β*	*p*	*β*	*p*
**OL1**											
Hexanoyl glycine / N-Acetyleucine	173.1052	7.05	Amino acid and proteins	0.00015	3.88E-03	0.00004	0.02	0.01	0.97	3.31	9.71E-07
N-Acetyleucine	173.1052	6.81	Amino acid and proteins	0.00016	2.75E-03	0.00003	0.05	0.55	0.12	2.66	5.19E-06
N-Acetyltyrosine	223.0843	4.96	Amino acid and proteins	0.00084	4.58E-06	0.00022	1.95E-04	0.50	0.05	1.58	5.28E-04
N-Acetyltryptophan	246.1004	7.45	Amino acid and proteins	0.00030	1.25E-03	0.00008	0.01	−0.01	0.98	1.07	3.39E-04
Ophthalmate	290.1343	1.42	Amino acid and proteins	−0.00024	3.16E-03	−0.00005	0.06	−0.61	0.01	−0.62	0.04
3-hydroxydodecanoyl carnitine	360.2741	11.69	Carnitines	−0.00022	0.05	−0.00005	0.20	−0.18	0.29	−0.98	2.61E-05
Riboflavin	377.1454	6.92	Vitamins	0.00046	6.26E-04	0.00010	0.02	0.44	3.27E-03	0.45	0.01
L-Thyroxine	777.6937	11.82	Amino acids and proteins/Hormones	−0.00013	0.04	−0.00002	0.32	−0.45	0.10	−0.96	0.01
**OL2a**											
Methoxycinnamic acid/3,4,5-trimethoxybenzaldehyde	161.0597	9.06	Phytochemicals	−0.00096	1.86E-03	−0.00014	0.04	0.22	0.23	1.08	0.01
5-Dodecenoic acid	181.1586	15.49	Fatty acid and lipids	−0.00012	0.02	−0.00002	0.30	−0.91	0.02	−1.84	3.28E-04
2-(2,3,4-trimethoxyphenyl)acetic acid	191.0700	8.90	Amino acid and proteins	−0.00029	0.02	−0.00003	0.48	−0.01	0.95	−0.73	1.10E-03
L-DOPA	197.0687	1.44	Amino acid and proteins	0.00016	0.01	0.00003	0.10	−0.12	0.68	1.04	0.03
Murideoxycholic acid/Ursodeoxycholate	197.1536	13.44	Bile acids	−0.00017	0.01	−0.00003	0.14	−0.41	0.18	−2.97	4.23E-08
5-Dodecenoic acid	199.1692	14.56	Fatty acid and lipids	−0.00014	0.02	−0.00002	0.24	−0.27	0.37	−2.77	7.78E-06
N-Acetyl-S-(3-hydroxypropyl-1-methyl)-L-cysteine	236.0947	5.42	Amino acid and proteins	−0.00062	0.02	−0.00010	0.24	0.04	0.56	−0.47	2.77E-06
5-Hydroxytryptophan	238.1185	2.28	Amino acid and proteins	−0.00024	4.92E-03	−0.00006	0.02	−0.83	9.48E-04	−2.47	6.86E-08
5′-Deoxyadenosine	251.1018	3.37	Nucleosides	0.00021	0.07	0.00007	0.05	0.04	0.80	0.65	1.15E-03
N-Acetyl-S-(3,4-dihydroxybutyl)-L-cysteine	252.0900	3.12	Amino acid and proteins	−0.00046	2.81E-03	−0.00007	0.14	0.00	0.998	−0.59	4.53E-04
2′-Deoxycytidine	260.1239	1.53	Nucleosides	0.00026	0.02	0.00005	0.15	0.04	0.82	1.21	7.22E-05
DGLA	289.2523	16.54	Fatty acid and lipids	−0.00012	0.03	−0.00004	0.04	−0.49	0.14	−1.44	3.71E-03
**OL2b**											
Indole-3-aldehyde	146.0601	5.05	Amino acid and proteins	0.00011	1.02E-03	0.00003	1.02E-03	0.35	0.54	2.04	0.01
Glutamine	147.0765	6.21	Amino acid and proteins	−0.00020	0.02	−0.00003	0.24	0.11	0.60	−1.47	3.97E-05
Quinaldic acid	174.0549	6.39	Amino acid and proteins	0.00024	0.04	0.00008	0.04	−0.24	0.22	0.88	8.37E-06
Quinaldic acid	174.0549	3.02	Amino acid and proteins	0.00015	0.07	0.00005	0.05	0.14	0.55	1.08	4.51E-04
Methylhippuric acid	193.0737	4.28	Xenobiotics	0.00040	1.59E-05	0.00010	7.98E-04	0.28	0.33	1.47	8.07E-04
10-Hydroxydecanoic acid	233.1130	1.46	Fatty acid and lipids	−0.00036	0.01	−0.00009	0.05	−0.15	0.22	−0.42	0.02
10-Hydroxydecanoic acid	233.1131	1.73	Fatty acid and lipids	−0.00030	1.64E-03	−0.00007	0.03	−0.45	0.02	0.12	0.65
2-amino-3-(4-hydroxy-3-methoxyphenyl)propanoic acid	234.0735	4.81	Amino acid and proteins	0.00024	3.13E-03	0.00005	0.06	0.48	0.04	0.65	0.04
Dioxacarb	262.0474	3.74	Xenobiotics	0.00023	0.06	0.00011	0.01	−0.15	0.28	0.46	0.03
6-Acetylmorphine/Naloxone	327.1469	12.14	Xenobiotics	0.00025	0.16	0.00012	0.03	−0.07	0.60	0.41	0.04
JWH-203	340.1476	8.95	Xenobiotics	0.00051	0.01	0.00014	0.02	0.04	0.65	0.61	4.91E-04
Glycocholate	488.2982	11.97	Bile acids	−0.00017	0.27	−0.00010	0.04	0.29	0.03	0.37	0.03

*DGLA* dihomo-gamma-linolenic acid, *L-DOPA* L-3,4-dihydroxyphenylalanine, *ETB* early term birth, *3HC* trans-3′-hydroxycotinine, *m/z* mass-to-charge ratio, *PTB* preterm birth.

^a^Metabolites reported were detected in at least 20% of newborn DBS samples. The OL1 annotation was reported in cases of multiple matches for the same metabolite.

^b^The beta coefficient represents the change in log_2_-transformed metabolite intensity per 1-unit increase in urinary creatinine-adjusted tobacco exposure biomarker.

^c^The beta coefficient represents the log odds change of early birth outcome with a one-unit increase in log_2_-transformed metabolite intensity.

### Newborn metabolic pathways associated with biomarkers of tobacco exposure and early birth outcomes

**Fig. 2:**
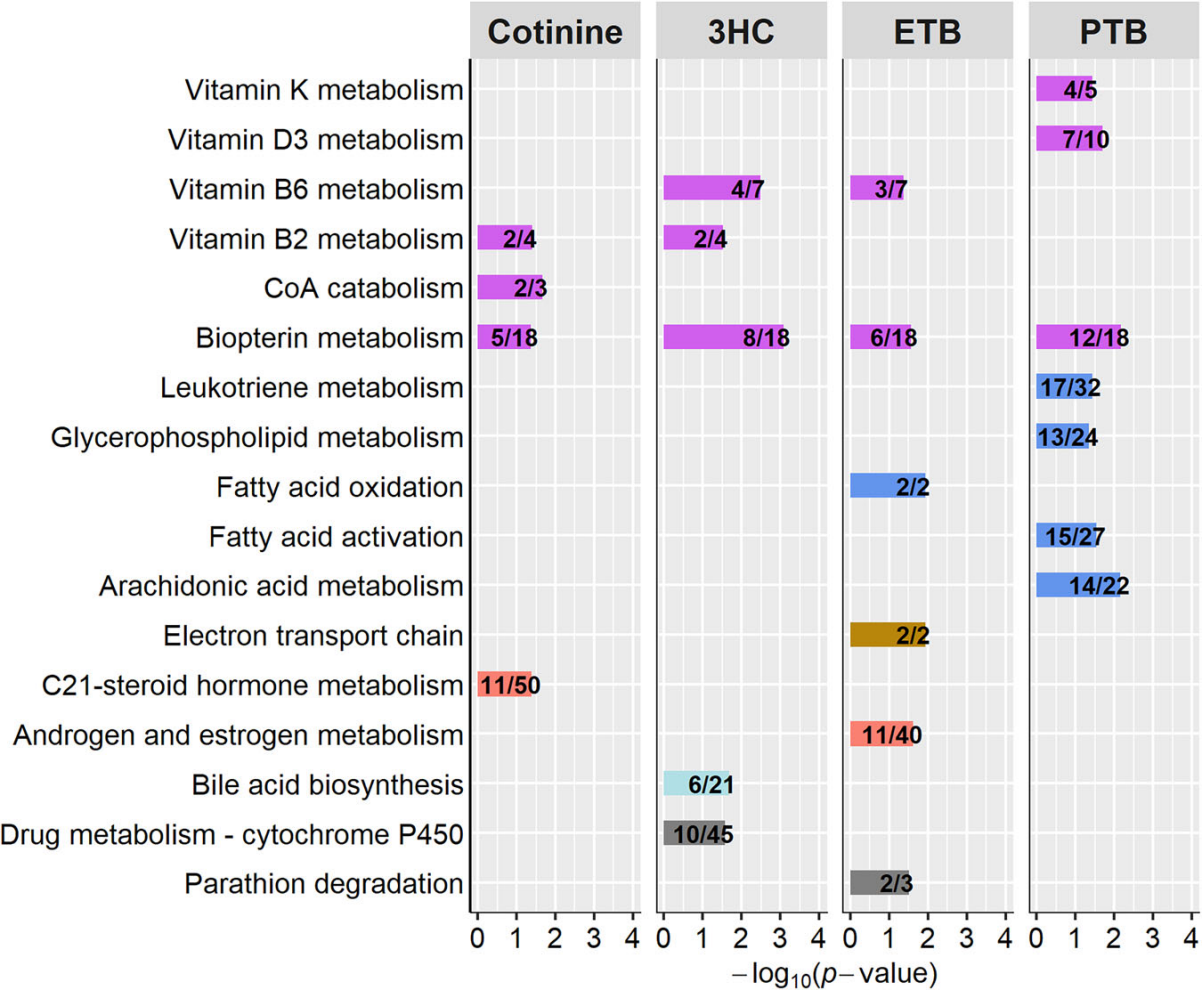
Newborn metabolic pathways associated (*p* value < 0.05) with prenatal tobacco exposure and/or early birth outcomes. Pathway enrichment analysis was conducted using *mummichog*. Metabolic pathways were grouped and colored by metabolic category: purple, vitamin and cofactor metabolism; blue, lipid and fatty acid metabolism; gold, energy metabolism; coral, hormone metabolism; light blue, bile acid metabolism; gray, drug metabolism/other metabolic pathways. The number of metabolites identified in the pathway and the total number of metabolites in the pathway are shown in each bar (metabolites overlapping/pathway size). CoA coenzyme A, ETB early term birth, 3HC trans-3′-hydroxycotinine, PTB preterm birth.

**Fig. 3:**
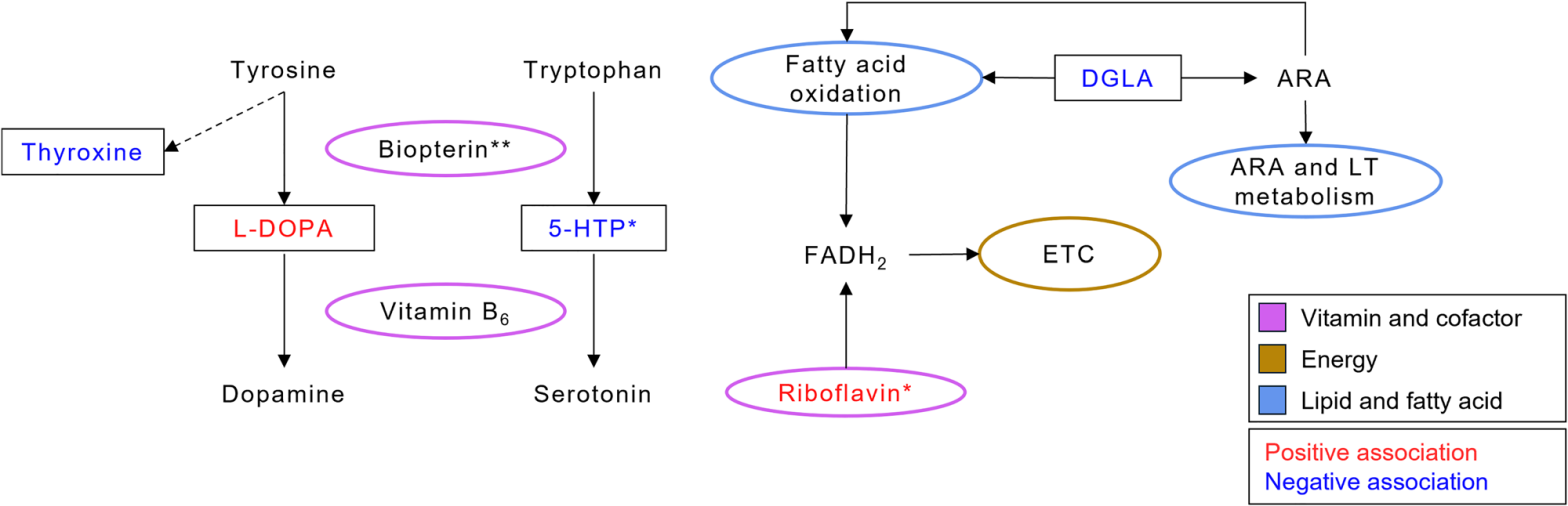
A summary of key significant metabolites and metabolic pathways in newborn DBS associated with prenatal tobacco exposure biomarkers and early birth outcomes. Metabolites significantly associated (*p* value < 0.05) with prenatal urinary tobacco exposure biomarkers and early birth are in boxes with positive associations represented in red and negative associations represented in blue. Metabolic pathways significantly associated with prenatal urinary tobacco exposure biomarkers and/or early birth are represented in circles colored by category. * denotes individual metabolites associated with all prenatal tobacco exposure biomarkers and early birth outcomes. ** denotes the metabolic pathway significantly associated with all prenatal tobacco exposure biomarkers and early birth outcomes. ARA arachidonic acid, DGLA dihomo-gamma-linolenic acid, L-DOPA 3,4-dihydroxyphenylalanine, ETC electron transport chain, FADH_2_ flavin adenine dinucleotide, 5-HTP 5-hydroxytryptophan, LT leukotriene.

Perturbations in the metabolism of vitamins and cofactors were characteristic of tobacco exposure biomarkers and early birth outcomes MWAS. Perturbation in the metabolism of the cofactor biopterin was significantly associated with both tobacco exposure biomarkers and both PTB and ETB (*p* value < 0.05) (Fig. 2). Vitamin B6 metabolism was also a common pathway associated with both 3HC and ETB. In addition to biopterin metabolism, vitamin B2 (riboflavin) metabolism was associated with both tobacco exposure biomarkers, and coenzyme A (CoA) catabolism was associated with cotinine. C21-steroid hormone metabolism was another metabolic pathway associated with cotinine concentrations. Bile acid biosynthesis was a significant metabolic pathway associated with only 3HC concentrations. For early birth outcomes, pathway enrichment results also included perturbations in lipid, fatty acid, and energy-related metabolic pathways in addition to perturbations in vitamin and cofactor metabolism (Fig. 2, Supplementary Table 6). In addition to biopterin and vitamin B6 metabolism, metabolic pathways associated with ETB included fatty acid oxidation, electron transport chain, androgen and estrogen metabolism, and parathion degradation. Metabolism of vitamins D3 and K was associated with PTB. Metabolites in leukotriene, glycerophospholipid, fatty acid activation, and arachidonic acid metabolic pathways were associated with PTB. A summary map of key metabolites and metabolic pathways that were linked to prenatal tobacco exposure and/or early birth is shown in Fig. 3.

### Sensitivity analysis

Metabolic pathway analyses were repeated at the threshold of FDR-corrected *q* value < 0.2 to reduce the number of potential false discoveries and identify consistent metabolic pathways. There were no common metabolic pathways between the tobacco biomarkers and adverse birth outcomes; however, biopterin metabolism remained significantly associated with PTB, and vitamin B2 metabolism remained significantly associated with both tobacco exposure biomarkers (Supplementary Fig. 6, Supplementary Table 7). The metabolism of vitamin D3 and K was consistently associated with PTB. Fatty acid-related pathways identified in the main analyses remained associated with ETB and PTB. The electron transport chain was similarly associated with ETB, and the tricarboxylic acid (TCA) cycle was an additional significant metabolic pathway associated with ETB. Ubiquinone biosynthesis was associated with ETB in the sensitivity analyses but not identified in the main analyses. Similarly, beta-alanine metabolism was associated with both tobacco exposure biomarkers. Hyaluronan metabolism and drug metabolism (other enzymes) were also identified as being associated with cotinine and 3HC, respectively.

## Discussion

Using untargeted HRM, we investigated the associations and newborn metabolic pathway perturbations between maternal urinary tobacco biomarker concentrations in early pregnancy and birth outcomes of the newborns among a subset of the Atlanta African American Maternal-Child Cohort. Maternal urinary cotinine and 3HC concentrations in early pregnancy and early birth outcomes were both associated with perturbations in newborn DBS metabolome, and particularly in biopterin metabolism. To our knowledge, this is the first study to use newborn DBS for metabolomics to elucidate the potential biological mechanisms associated with both prenatal tobacco exposure and early birth.

Biopterin metabolism was associated with both maternal urinary cotinine and 3HC concentrations as well as ETB and PTB. Biopterin, in the form of tetrahydrobiopterin (BH4), is an essential cofactor required for various enzymes, including tryptophan and tyrosine hydroxylases^44^. Further supporting this finding, we also found that the products of tryptophan hydroxylase and tyrosine hydroxylase activity (5-hydroxytryptophan and L-DOPA, respectively) were associated with at least one of the tobacco biomarkers and early birth outcomes. The production of these metabolites is therefore dependent on efficient biopterin biosynthesis and recycling^44^. These metabolites are precursors for critical neurotransmitters. The precursor for serotonin is 5-hydroxytryptophan, and L-DOPA is the immediate precursor for dopamine^44,45^. Animal studies have shown that the placenta is a producer of serotonin and dopamine for the developing fetus^46^. In the present study, we found an inverse, significant association between 5-hydroxytryptophan and both tobacco biomarkers and early birth. Decreased 5-hydroxytryptophan signal intensities with early birth outcomes are aligned with another study that found decreased tryptophan hydroxylase gene expression in PTB placenta compared to full-term births^47^. L-DOPA was positively associated with cotinine concentrations, which may reflect the dopaminergic response to nicotine. The role of dopamine in promoting nicotine dependence and addiction is well established; however, further studies are needed to understand the mechanism linking fetal dopamine production to early birth^48^.

Impaired newborn biopterin metabolism due to tobacco exposure may also extend to disrupted nitric oxide (NO) production by NO synthase (NOS), which utilizes BH4 as a cofactor and plays a vital role in placental development^44^. Production of nitric oxide by NOS regulates trophoblast invasion, vascular remodeling, and blood flow, which are essential factors of normal placental development in early pregnancy^49^. Several studies have linked tobacco smoke to BH4 depletion and subsequent disrupted NO production. In vitro studies have shown that cigarette smoke extract exposure reduced expression of the rate-limiting enzyme in BH4 synthesis, depleted BH4 levels, and decreased NO production^50^. In an animal model, endothelial cell BH4 deficiency resulted in increased systolic blood pressure during gestation, reduced placental size, and fetal growth restriction in pups^51^. In the same study, human umbilical vein endothelial cells from women who had gestational hypertension showed reduced BH_4_ levels and endothelial NOS activity compared to those from women who did not have gestational hypertension^51^. In the absence of BH4, NOS can produce reactive oxygen species, resulting in increased oxidative stress^50,52^. Similarly, in the context of early pregnancy, maternal prenatal smoking may increase the risk of early birth by depleting BH4 and leading to reduced endogenous NO availability and abnormal placental development, compounded with elevated oxidative stress in the fetal-placental unit. This study identified more metabolomic signals associated with PTB compared to ETB, including after FDR correction. Aside from perturbations in biopterin metabolism, there were mostly distinct metabolic pathway perturbations between ETB and PTB. Our findings support other studies indicating that while there are some shared determinants for ETB and PTB, there may be other distinct etiologies or determinants for PTB^53,54^.

Metabolism of vitamins B2 and B6 was associated with at least one tobacco biomarker, underscoring the role of vitamins in modulating the inflammatory and oxidative stress effects of tobacco. Vitamin B6 is a coenzyme involved in the synthesis of both dopamine and serotonin, as it supports the conversions of 5-hydroxytryptophan and L-DOPA to serotonin and dopamine, respectively^45^. Riboflavin in the active forms of flavin mononucleotide (FMN) and flavin adenine dinucleotide (FAD) are common coenzymes in multiple metabolic reactions that are essential to energy metabolism and antioxidant defense capacity^55^. The positive association between newborn riboflavin and tobacco exposure biomarkers may reflect decreased synthesis of FAD and FMN, which are also involved in NOS activity^52^ and the regeneration of the antioxidant glutathione^55^. Previous studies have shown impaired glutathione balance in mothers who smoked during pregnancy and in their newborns^56^. Perturbations in vitamins B2 and B6 metabolic pathways in newborns may indicate elevated oxidative stress during gestation induced by prenatal tobacco exposure.

In the present study, thyroxine, a form of the thyroid hormone, was inversely associated with cotinine and PTB, implicating a possible impairment in thyroid function. The thyroid hormone regulates multiple critical processes, including basal metabolic rate and fetal brain development^57^. Smoking during pregnancy has been shown to impair transcription of key genes in fetal thyroid development^58^. The results of the association of tobacco exposure during pregnancy on free thyroxine concentrations in the fetal compartment are mixed, with some studies revealing no association, and other studies showing a gestational age-dependent association^58–60^. Here, newborn blood thyroxine was negatively associated with maternal urinary cotinine concentrations. This difference may be due to the stage of fetal development, hormone form (free vs total thyroxine), biospecimen analyzed (cord serum vs infant blood), and length of tobacco exposure time. As the fetal thyroid gland develops early in gestation^61^, it may be particularly sensitive to tobacco exposure in early pregnancy; further studies are warranted to investigate the role of fetal thyroid hormone in PTB.

PTB was associated with metabolites in the arachidonic acid and leukotriene metabolic pathways that act as bioactive lipid mediators in inflammatory processes^62^. Inflammation and oxidative stress are drivers of abnormal placental development leading to adverse conditions for the developing fetus, including hypoxia and restricted nutrient supply^14^. Leukotrienes derived from arachidonic acid have immunomodulatory properties, including immune activation and systemic inflammation^62^. Leukotriene metabolism was associated with urinary cotinine concentrations, which may reflect immunomodulatory actions. Specific prostaglandins derived from arachidonic acid are involved in the normal process of parturition^63^ and used pharmacologically for labor induction^64^. Targeted metabolomics studies of the relationship between prenatal tobacco smoke exposure and metabolome in the fetal compartment using cord blood have identified changes in lipids, including sphingomyelin and phosphatidylcholine metabolite categories^24,26^. Our findings linking perturbations in arachidonic acid and leukotriene metabolism in newborns to maternal smoking during pregnancy are consistent with another HRM study of newborn DBS^28^.

Previous metabolomics studies of tobacco exposure in pregnancy have shown a pattern of metabolic perturbations related to vitamins with known antioxidant properties. However, there may be varied responses of different vitamin metabolic pathways in offspring compared to their mothers. Studies focused on maternal metabolomic changes with tobacco smoke exposure identified metabolism of vitamins A, C, and E^20,25^. For the newborn or fetal compartment metabolome, vitamin A metabolism was also linked to maternal tobacco smoke exposure during pregnancy^25,28^. In contrast to these studies, the present study did not find significant perturbations in vitamins A, C, or E metabolic pathways in newborns associated with tobacco smoke exposure, but rather metabolic perturbations with vitamin B2 and B6. These differences in metabolomics results may be due to data collection for tobacco exposure in early pregnancy compared to mid- or late pregnancy or in the neonatal period. Given the role of vitamin B2 and B6 in supporting antioxidant systems^55,65^, this study further supports the prominence of vitamins with antioxidant roles in counteracting the deleterious effects of tobacco exposure.

Our major study strength is the focus on only African American pregnant women and their newborns, a population that is understudied despite concerning persistent disparities in birth outcomes^7^. Other studies have used self-reported maternal smoking to estimate tobacco smoke exposure, which may be prone to bias^26,27,66^. Here, our analysis utilized targeted assessment of established and validated biomarkers of tobacco smoke exposure, which allowed for identification of tobacco smoke exposure in participants who reported no tobacco use or had no tobacco use in their medical record but had detectable urinary nicotine metabolites^12,67^. An additional strength of this analysis is the use of HRM for newborn DBS captured within 24–48 h of birth to study the effect of ETB and PTB on the newborn metabolome as an indicator of the in utero metabolome. Our study also has several limitations. Our study focused on tobacco smoke exposure at a single time point in early pregnancy; further studies are needed to assess metabolic perturbations linked to tobacco smoke exposure at multiple time points in gestation. Additionally, tobacco smoke may also interact with other environmental chemicals that may mask the health effects of tobacco. While we focused our analysis on a single, but major, environmental exposure, further studies are needed to consider combinations of environmental exposures and stressors.

Using untargeted HRM, we identified metabolic pathways and metabolites associated with tobacco exposure biomarkers and early birth in African American mother-newborn pairs. Our study showed perturbations in biopterin metabolism as a potential mechanism linking prenatal tobacco smoke exposure and early birth. Insight into these metabolic changes can inform future studies of targeted interventions to reduce early births due to tobacco smoke exposure in this population.

## Supplementary information

Supplementary Information

Description of Additional Supplementary Files

Supplementary Data

## Data Availability

The raw and processed metabolomics data from this study have been archived in the Metabolomics Workbench (https://www.metabolomicsworkbench.org/, Study ID ST002692) through the UNC HHEAR Laboratory. Exposure and outcome data are not publicly available due to data privacy regulations. Source data for the main figures presented are found in the Supplementary Data file.

## Code availability

Pathway enrichment analyses were performed using *mummichog* (version 2.7). All analysis scripts used in this study are available from the corresponding author upon reasonable request.
