# Supplementary material for: Newborn metabolomic perturbations associated with prenatal tobacco smoke exposure and early birth
Source: Commun Med (Lond). 2026 Apr 1;6:321. doi: 10.1038/s43856-026-01534-5 (PMC13230697; doi:10.1038/s43856-026-01534-5)
Supplement: Supplementary file 1 — Supplementary Information [file 43856_2026_1534_MOESM1_ESM.pdf]

Supplementary Information

Newborn metabolomic perturbations associated with prenatal tobacco smoke exposure and early birth

Paula-Dene C. Nesbeth [1], Xiajie Lyu [1], Anne L. Dunlop [2], Youran Tan [1], Dana Boyd Barr [1], Volha Yakimavets [1], Parinya Panuwet [1], Mengyuan Ren [1], Stephanie M. Eick [1], Blake R. Rushing [3,4], Susan L. McRitchie [4], Susan Sumner [3,4], P. Barry Ryan [1], Elizabeth J. Corwin [5], Dean P. Jones [6], Donghai Liang [1]

[1] Gangarosa Department of Environmental Health, Rollins School of Public Health, Emory University, Atlanta, GA, USA.
[2] Department of Gynecology and Obstetrics, School of Medicine, Emory University, Atlanta, GA, USA.
[3] Department of Nutrition, University of North Carolina at Chapel Hill, Chapel Hill, NC, USA.
[4] Nutrition Research Institute, University of North Carolina at Chapel Hill, Kannapolis, NC, USA.
[5] School of Nursing, Columbia University, New York, NY, USA.
[6] School of Medicine, Emory University, Atlanta, GA, USA.

Corresponding Author:
Donghai Liang, PhD, MPH
Gangarosa Department of Environmental Health
Emory University Rollins School of Public Health
1518 Clifton Rd, Rm 2037
Atlanta, GA 30322, USA
donghai.liang@emory.edu

Supplementary Table 1. Maternal unadjusted and creatinine-adjusted urine cotinine and 3HC concentrations among all participants

	**All Participants (*n* = 269)**	**No tobacco use [a] (*n* = 225)**	**Tobacco use [a] (*n* =44)**
Cotinine			
Above LOD (*n*, %)	205 (76.2)	161 (71.6)	44 (100)
Unadjusted (ng/mL urine)	12.50 (11.12)	6.8 (7.24)	282.26 (7.15)
Adjusted (μg/g creatinine)	7.44 (10.90)	4.04 (7.0)	168.95 (7.22)
3HC			
Above LOD (*n*, %)	208 (77.3)	164 (72.9)	44 (100)
Unadjusted (ng/mL urine)	25.03 (14.58)	12.86 (9.60)	754.87 (7.21)
Adjusted (μg/g creatinine)	14.89 (13.81)	7.64 (8.88)	451.82 (7.16)

Values reported are geometric mean (geometric SD).

[a] Tobacco use in the last month prior to study visit or anytime in pregnancy. 3HC, trans-3′-hydroxycotinine; LOD, limit of detection.

Supplementary Table 2. Overlapping significant metabolomic signals across all metabolome-wide association studies (*p*-value < 0.05)

	**Tobacco exposure biomarker**	
**Early Birth Outcome**	Cotinine	3HC
ETB	116	84
PTB	348	236

ETB, early term birth; 3HC, trans-3′-hydroxycotinine; PTB, preterm birth.

Supplementary Table 3. Overlapping significant metabolomic signals across all metabolome-wide association studies (FDR-corrected *q*-value < 0.2)

	**Tobacco exposure biomarker**	
**Early Birth Outcome**	Cotinine	3HC
ETB	4	2
PTB	78	34

ETB, early term birth; FDR, false discovery rate correction of multiple testing using Benjamini-Hochberg procedure; 3HC, trans-3′-hydroxycotinine; PTB, preterm birth.

Supplementary Table 4. Newborn dried blood spot metabolites associated with prenatal tobacco exposure biomarkers (creatinine adjusted) and early birth outcomes (FDR-corrected *q*-values using the Benjamini-Hochberg procedure)

Metabolite[a]	m/z	rt (min)	Category	Cotinine[b]		3HC[b]		ETB[c]		PTB[c]	
				β	***q***	***β***	***q***	***β***	***q***	***β***	***q***
**OL1**											
Hexanoyl glycine / N-Acetyleucine	173.1052	7.05	Amino acid and proteins	0.00015	0.21	0.00004	0.51	0.01	0.99	3.31	0.0001
N-Acetyleucine	173.1052	6.81	Amino acid and proteins	0.00016	0.19	0.00003	0.68	0.55	0.62	2.66	0.0004
N-Acetyltyrosine	223.0843	4.96	Amino acid and proteins	0.00084	0.01	0.00022	0.07	0.50	0.49	1.58	0.01
N-Acetyltryptophan	246.1004	7.45	Amino acid and proteins	0.00030	0.14	0.00008	0.35	-0.01	1	1.07	0.01
Ophthalmate	290.1343	1.42	Amino acid and proteins	-0.00024	0.19	-0.00005	0.68	-0.61	0.35	-0.62	0.16
3-hydroxydodecanoyl carnitine	360.2741	11.69	Carnitines	-0.00022	0.50	-0.00005	0.84	-0.18	0.73	-0.98	0.001
Riboflavin	377.1454	6.92	Vitamins	0.00046	0.10	0.00010	0.57	0.44	0.27	0.45	0.09
L-Thyroxine	777.6937	11.82	Amino acid and proteins / Hormones	-0.00013	0.48	-0.00002	0.90	-0.45	0.60	-0.96	0.05
**OL2a**											
Methoxycinnamic acid / 3,4,5-trimethoxybenzaldehyde	161.0597	9.06	Phytochemicals	-0.00096	0.16	-0.00014	0.63	0.22	0.70	1.08	0.08
5-Dodecenoic acid	181.1586	15.49	Fatty acid and lipids	-0.00012	0.37	-0.00002	0.89	-0.91	0.40	-1.84	0.01
2-(2,3,4-trimethoxyphenyl)acetic acid	191.0700	8.90	Amino acid and proteins	-0.00029	0.40	-0.00003	0.94	-0.01	0.99	-0.73	0.02
L-DOPA	197.0687	1.44	Amino acid and proteins	0.00016	0.25	0.00003	0.75	-0.12	0.91	1.04	0.13

Murideoxycholic acid / Ursodeoxycholate	197.1536	13.44	Bile acids	-0.00017	0.27	-0.00003	0.79	-0.41	0.65	-2.97	0.00003
5-Dodecenoic acid	199.1692	14.56	Fatty acid and lipids	-0.00014	0.35	-0.00002	0.86	-0.27	0.78	-2.77	0.0005
N-Acetyl-S-(3-hydroxypropyl-1-methyl)-L-cysteine	236.0947	5.42	Amino acid and proteins	-0.00062	0.37	-0.00010	0.86	0.04	0.86	-0.47	0.0003
5-Hydroxytryptophan	238.1185	2.28	Amino acid and proteins	-0.00024	0.23	-0.00006	0.54	-0.83	0.16	-2.47	0.00004
5'-Deoxyadenosine	251.1018	3.37	Nucleosides	0.00021	0.56	0.00007	0.65	0.04	0.95	0.65	0.02
N-Acetyl-S-(3,4-dihydroxybutyl)-L-cysteine	252.0900	3.12	Amino acid and proteins	-0.00046	0.19	-0.00007	0.80	0.00	1.00	-0.59	0.01
2'-Deoxycytidine	260.1239	1.53	Nucleosides	0.00026	0.37	0.00005	0.80	0.04	0.96	1.21	0.003
DGLA	289.2523	16.54	Fatty acid and lipids	-0.00012	0.43	-0.00004	0.63	-0.49	0.63	-1.44	0.04
**OL2b**											
Indole-3-aldehyde	146.0601	5.05	Amino acid and proteins	0.00011	0.13	0.00003	0.14	0.35	0.85	2.04	0.06
Glutamine	147.0765	6.21	Amino acid and proteins	-0.00020	0.38	-0.00003	0.86	0.11	0.87	-1.47	0.002
Quinaldic acid	174.0549	6.39	Amino acid and proteins	0.00024	0.48	0.00008	0.65	-0.24	0.68	0.88	0.001
Quinaldic acid	174.0549	3.02	Amino acid and proteins	0.00015	0.56	0.00005	0.65	0.14	0.85	1.08	0.01
Methylhippuric acid	193.0737	4.28	Xenobiotics	0.00040	0.02	0.00010	0.13	0.28	0.75	1.47	0.01
10-Hydroxydecanoic acid	233.1130	1.46	Fatty acid and lipids	-0.00036	0.33	-0.00009	0.66	-0.15	0.68	-0.42	0.10
10-Hydroxydecanoic acid	233.1131	1.73	Fatty acid and lipids	-0.00030	0.15	-0.00007	0.60	-0.45	0.40	0.12	0.84
2-amino-3-(4-hydroxy-3-methoxyphenyl)propanoic acid	234.0735	4.81	Amino acid and proteins	0.00024	0.19	0.00005	0.68	0.48	0.47	0.65	0.17
Dioxacarb	262.0474	3.74	Xenobiotics	0.00023	0.54	0.00011	0.35	-0.15	0.72	0.46	0.13

6-Acetylmorphine / Naloxone	327.1469	12.14	Xenobiotics	0.00025	0.70	0.00012	0.58	-0.07	0.88	0.41	0.16
JWH-203	340.1476	8.95	Xenobiotics	0.00051	0.24	0.00014	0.54	0.04	0.89	0.61	0.01
Glycocholate	488.2982	11.97	Bile acids	-0.00017	0.80	-0.00010	0.63	0.29	0.45	0.37	0.14

3HC, trans-3′-hydroxycotinine; DGLA, dihomo-gamma-linolenic acid; L-DOPA, L-3,4-dihydroxyphenylalanine; ETB, early term birth; m/z, mass-to-charge ratio; PTB, preterm birth.

[a] Metabolites listed were detected in at least 20% of newborn DBS samples. The OL1 annotation was reported in cases of multiple matches for the same metabolite.

[b] The beta coefficient represents the change in log_2-transformed metabolite intensity per 1-unit increase in urinary creatinine-adjusted tobacco exposure biomarker.

[c] The beta coefficient represents the log odds change of early birth outcome with a one-unit increase in log_2-transformed metabolite intensity.

Supplementary Table 5. Associations of riboflavin and 5-hydroxytryptophan with cotinine, 3HC, ETB, and PTB by marijuana use.

	**Marijuana use [a] (*n* = 100)**		**No Marijuana use (*n* = 169)**	
	**β**	***p***	**β**	***p***
**Riboflavin (m/z: 377.1454; time: 6.92)**				
COT	7.21E-04	1.57E-04	2.80E-04	0.158
3HC	2.09E-04	0.003	3.41E-05	0.548
ETB	0.363	0.179	0.423	0.025
PTB	0.586	0.066	0.432	0.067
**5-hydroxytryptophan (m/z: 238.1185; time: 2.28)**				
COT	-4.02E-04	0.001	-5.02E-06	0.967
3HC	-1.26E-04	0.005	-1.10E-05	0.749
ETB	-3.674	2.92E-05	-0.304	0.291
PTB	-3.721	0.001	-2.317	4.45E-05

[a] Marijuana use in the last month or anytime in pregnancy. β and *p*-value determined by multivariable linear regression (for COT and 3HC) or logistic regression (for ETB and PTB) adjusting for covariates. COT, cotinine; ETB, early term birth; 3HC, trans-3′-hydroxycotinine; PTB, preterm birth.

Supplementary Table 6. Metabolic pathways associated with prenatal tobacco exposure and/or early birth outcomes (p-value < 0.05)[a]

MWAS	Pathway	Overlap size	Pathway size	Overlap %	*p*-value
COT	CoA catabolism	2	3	67	0.022
COT	Vitamin B2 metabolism	2	4	50	0.040
COT	C21-steroid hormone metabolism	11	50	22	0.041
COT	Biopterin metabolism	5	18	28	0.043
3HC	Biopterin metabolism	8	18	44	0.001
3HC	Vitamin B6 metabolism	4	7	57	0.003
3HC	Bile acid biosynthesis	6	21	29	0.021
3HC	Drug metabolism - cytochrome P450	10	45	22	0.027
3HC	Vitamin B2 metabolism	2	4	50	0.031
ETB	Electron transport chain	2	2	100	0.012
ETB	Fatty acid oxidation	2	2	100	0.012
ETB	Androgen and estrogen metabolism	11	40	28	0.024
ETB	Biopterin metabolism	6	18	33	0.027
ETB	Parathion degradation	2	3	67	0.031
ETB	Vitamin B6 metabolism	3	7	43	0.043
PTB	Biopterin metabolism	12	18	67	0.007
PTB	Arachidonic acid metabolism	14	22	64	0.007
PTB	Vitamin D3 metabolism	7	10	70	0.020
PTB	Fatty acid activation	15	27	56	0.028
PTB	Vitamin K metabolism	4	5	80	0.037
PTB	Leukotriene metabolism	17	32	53	0.037
PTB	Glycerophospholipid metabolism	13	24	54	0.044

[a] Metabolomic signal significance threshold for pathway enrichment analysis was p-value < 0.05.
CoA, coenzyme A; COT, cotinine; ETB, early term birth; 3HC, trans-3′-hydroxycotinine; MWAS, metabolome-wide association studies; PTB, preterm birth.

Supplementary Table 7. Metabolic pathways associated with prenatal tobacco exposure and/or early birth outcomes (p-value < 0.05)[a]

MWAS	Pathway	Overlap size	Pathway size	Overlap %	p-value
COT	C21-steroid hormone metabolism	6	50	12	0.0004
COT	Hyaluronan metabolism	1	3	33	0.023
COT	Vitamin B2 metabolism	1	4	25	0.032
COT	Beta-Alanine metabolism	1	5	20	0.039
3HC	Vitamin B2 metabolism	1	4	25	0.016
3HC	Beta-Alanine metabolism	1	5	20	0.020
3HC	Drug metabolism - other enzymes	1	7	14	0.028
ETB	Androgen and estrogen metabolism	6	40	15	0.0001
ETB	Electron transport chain	2	2	100	0.0003
ETB	Fatty acid oxidation	2	2	100	0.0003
ETB	TCA cycle	2	6	33	0.002
ETB	Ubiquinone biosynthesis	2	12	17	0.006
PTB	Biopterin metabolism	12	18	67	0.009
PTB	Arachidonic acid metabolism	14	22	64	0.010
PTB	Vitamin D3 metabolism	7	10	70	0.025
PTB	Fatty acid activation	15	27	56	0.040
PTB	Vitamin K metabolism	4	5	80	0.042
PTB	Bile acid biosynthesis	12	21	57	0.044
PTB	Leukotriene metabolism	17	32	53	0.048

[a] Metabolomic signal significance threshold for pathway enrichment analysis was FDR-corrected q-value < 0.2. COT, cotinine; ETB, early term birth; FDR, false discovery rate; 3HC, trans-3′-hydroxycotinine; MWAS, metabolome-wide association studies; PTB, preterm birth, TCA, tricarboxylic acid.

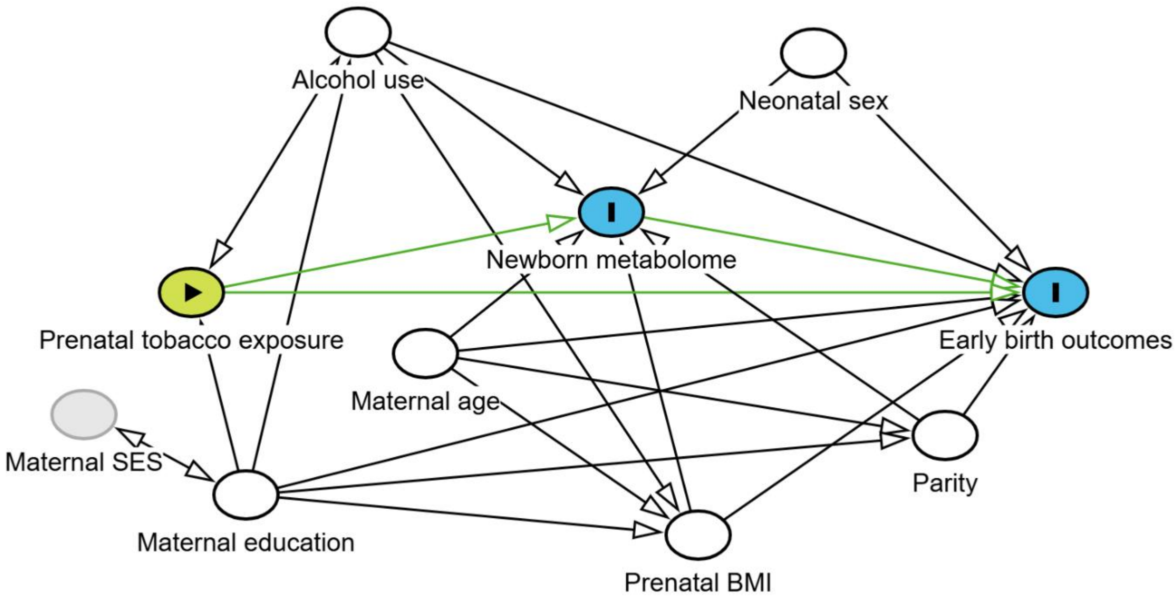

Supplementary Fig 1. A directed acyclic graph used for selection of potential confounders. Figure created using *dagitty* (https://www.dagitty.net/dags.html). BMI, body mass index; SES, socioeconomic status

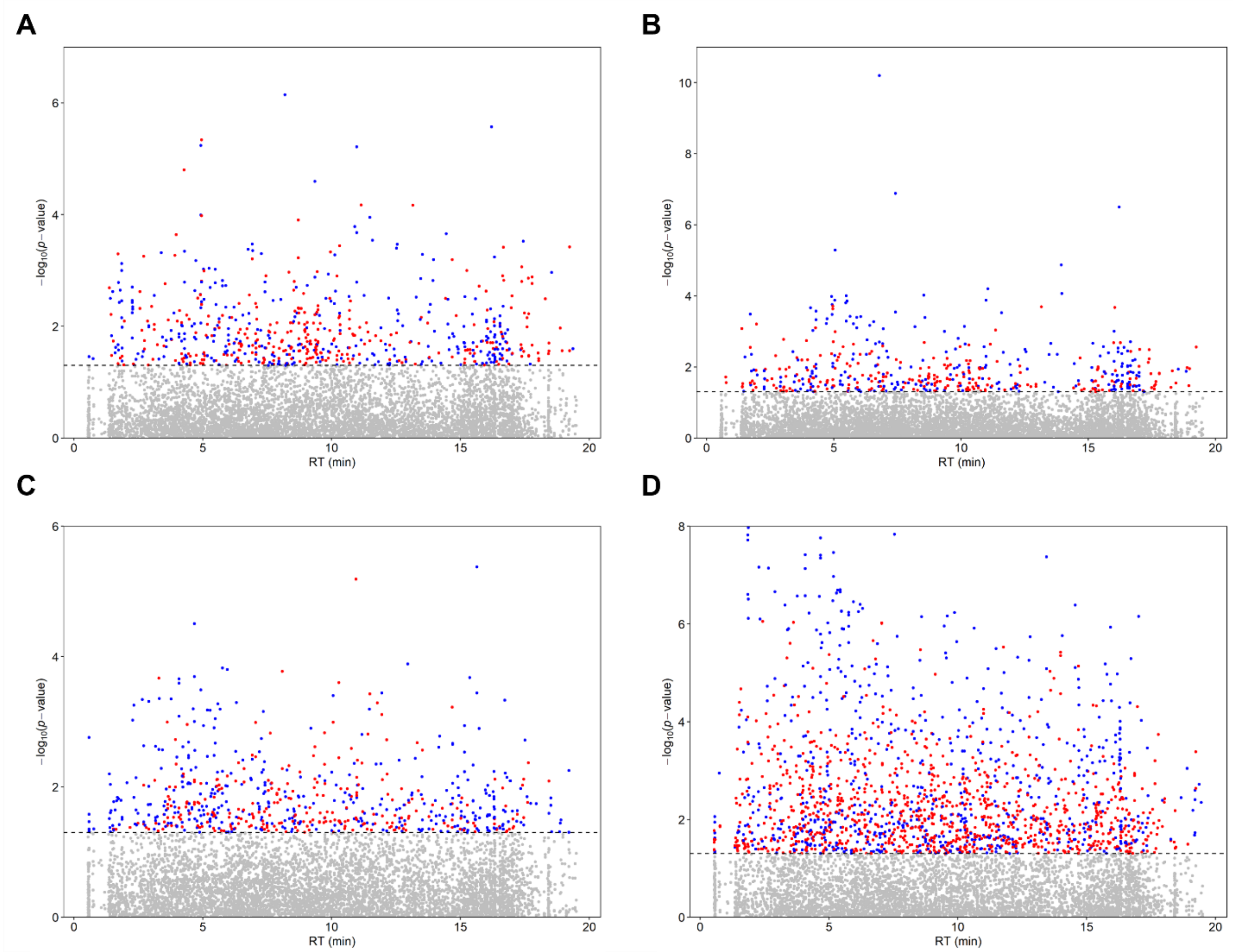

Supplementary Fig. 2. Manhattan plots of newborn DBS metabolomic signals associations with prenatal tobacco exposure biomarkers, cotinine (A) and 3HC (B), and early birth outcomes, ETB (C) and PTB (D). Associations between each signal and the exposures or outcomes were determined by multivariable linear regression or logistic regression adjusting for covariates. Dashed line represents the -log10(*p*-value) corresponding to *p*-value = 0.05. Points above the dashed line represent significant positive (in red) or a significant negative association (in blue). DBS, dried blood spot, ETB, early term birth; 3HC, trans-3′-hydroxycotinine; PTB, preterm birth.

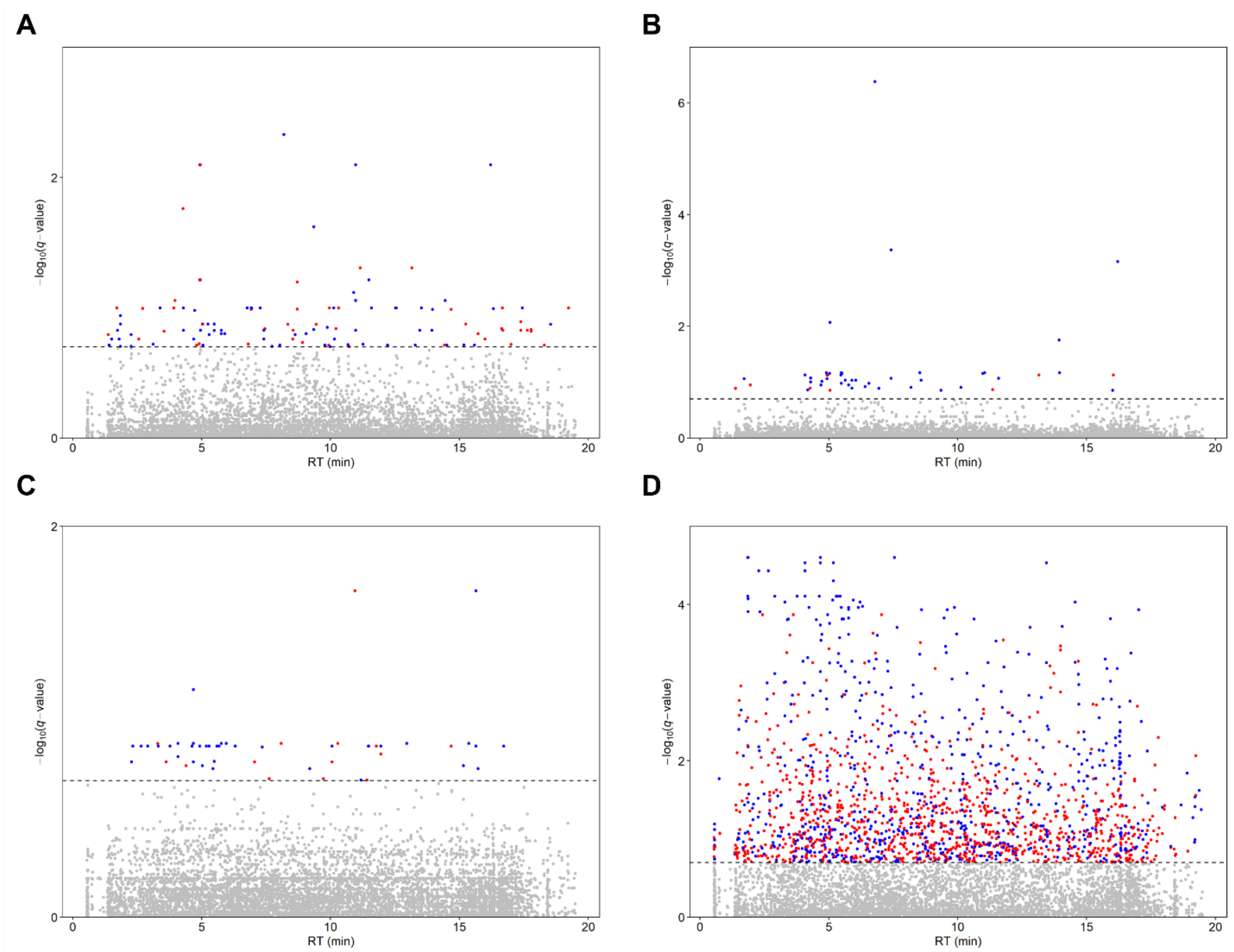

Supplementary Fig. 3. Manhattan plots of newborn DBS metabolomic signals associations with prenatal tobacco exposure biomarkers, cotinine (A) and 3HC (B), and early birth outcomes, ETB (C) and PTB (D). Associations between each signal and the exposures or outcomes were determined by multivariable linear regression or logistic regression adjusting for covariates. FDR correction was performed using the Benjamini-Hochberg procedure. Dashed line represents the -log$_{10}$(FDR-corrected q-value) corresponding to q-value = 0.2. Points above the dashed line represent significant positive (in red) or a significant negative association (in blue). DBS, dried blood spot, ETB, early term birth; 3HC, trans-3′-hydroxycotinine; PTB, preterm birth.

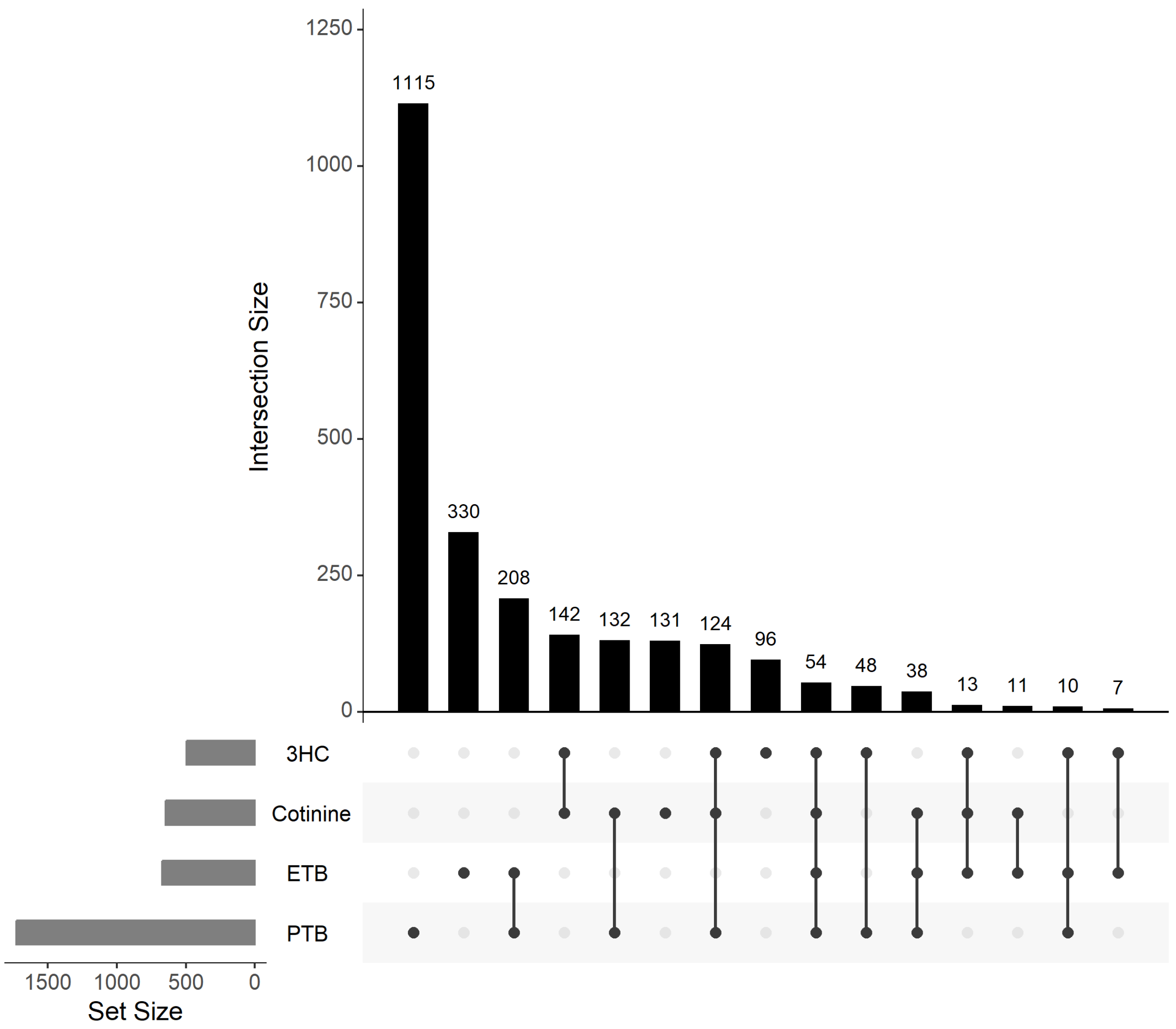

Supplementary Fig. 4. UpSet plot showing overlapping significant metabolomic signals associated with prenatal tobacco exposure biomarkers and early birth outcomes (two-sided *p*-value < 0.05). Significant metabolomic signals for each tobacco exposure biomarkers and early birth outcome were identified by multivariable linear regression or logistic regression adjusting for covariates. The number of overlapping metabolomic signals in each intersection are shown in the bar graph above. Black points with no lines represent metabolomic signals unique to the corresponding tobacco exposure biomarker or early birth outcome. ETB, early term birth; 3HC, trans-3′-hydroxycotinine; PTB preterm birth.

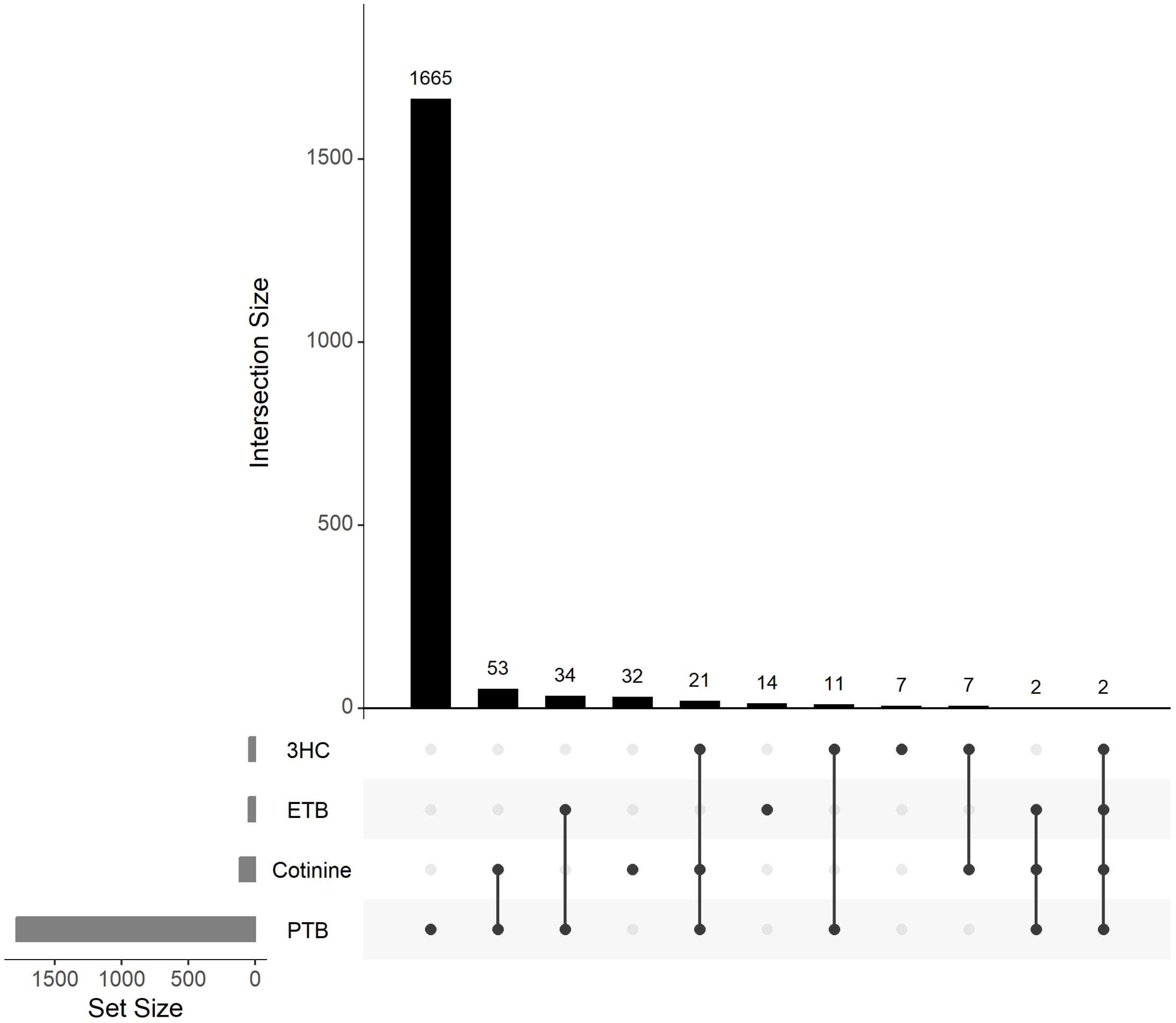

Supplementary Fig. 5. UpSet plot showing the overlapping significant metabolomic associations with prenatal tobacco exposure biomarkers and early birth outcomes (FDR-corrected *q*-value < 0.2). Significant metabolomic signals for each tobacco exposure biomarkers and early birth outcome were identified by multivariable linear regression or logistic regression adjusting for covariates. FDR correction was performed using the Benjamini-Hochberg procedure. The number of overlapping metabolomic signals in each intersection are shown in the bar graph above. Black points with no lines represent metabolomic signals unique to the corresponding tobacco exposure biomarker or early birth outcome. ETB, early term birth; 3HC, trans-3′-hydroxycotinine; PTB preterm birth.

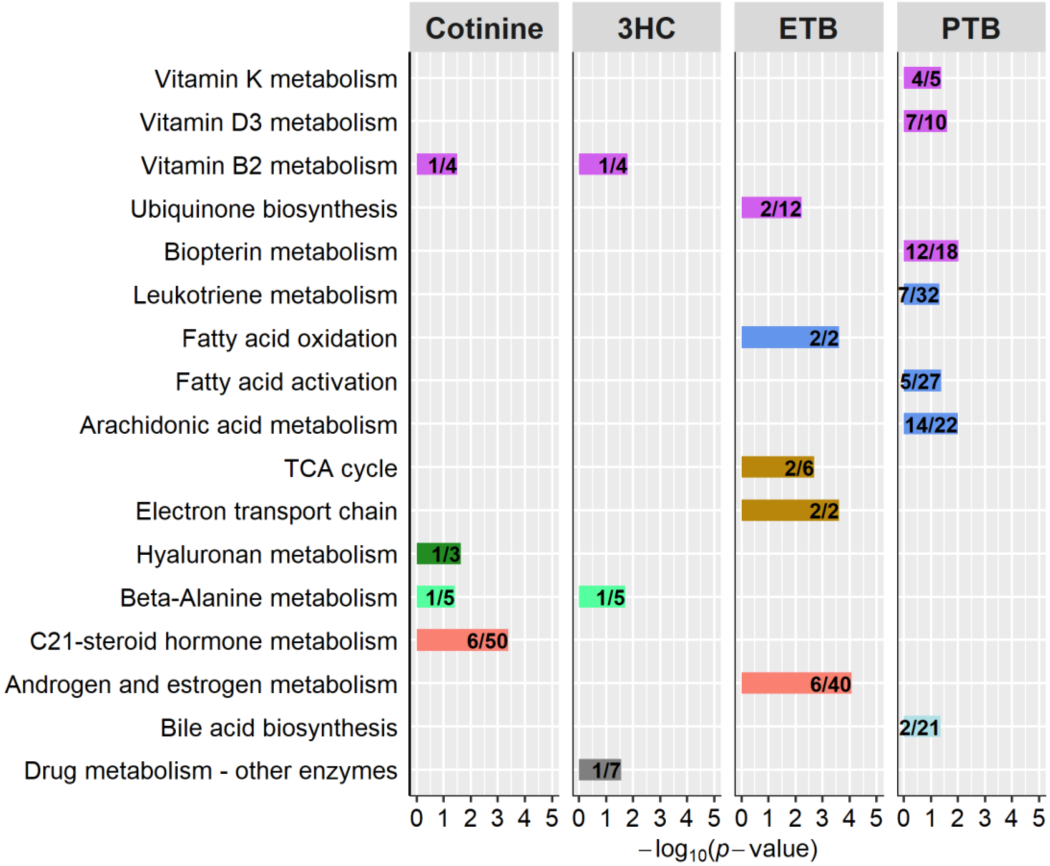

Supplementary Fig. 6. Newborn metabolic pathways associated (p-value < 0.05) with prenatal tobacco biomarkers and/or early birth outcomes. Metabolomic features with q-values < 0.2 after FDR correction using the Benjamini-Hochberg procedure were included in the pathway analysis. Pathway enrichment analysis was conducted using *mummichog*. Metabolic pathways were grouped and colored by metabolic category: purple, vitamin and cofactor metabolism; blue, lipid and fatty acid metabolism; gold, energy metabolism; dark green, carbohydrate metabolism; light green, amino acid metabolism; coral, hormone metabolism; light blue, bile acid metabolism; gray, drug metabolism/other metabolic pathways. The numbers of metabolites identified in the pathway and total metabolites within the pathway are shown in each bar (number of metabolites identified/number of total metabolites within the pathway). DBS, dried blood spot; ETB, early term birth; 3HC, trans-3′-hydroxycotinine; PTB preterm birth; TCA, tricarboxylic acid.
